# Dimethyl malonate alleviates obstructive nephropathy by enhancing renal metabolism and inhibiting kidney oxidative stress and inflammation

**DOI:** 10.3389/fphar.2025.1530635

**Published:** 2025-06-10

**Authors:** Wei Zhang, Changde Fu, Jinjin Lai, Jun Xin, Wenbin Zhang

**Affiliations:** Department of Urology, Quanzhou First Hospital Affiliated to Fujian Medical University, Quanzhou, China

**Keywords:** obstructive nephropathy, dimethyl malonate, mitochondria, oxidative stress, inflammation

## Abstract

**Introduction:**

Obstructive nephropathy is a leading cause of renal injury and fibrosis. Mitochondrial dysfunction represents a hallmark of obstructive nephropathy, a condition that leads to metabolic aberrations, succinate accumulation, reactive oxygen species (ROS) overproduction, tubular damage, and kidney inflammation. Succinate dehydrogenase (SDH) is central to mitochondrial metabolism and targeting SDH with dimethyl malonate (DMM) has been shown to be effective in treating renal ischemia-reperfusion (IR) injury in the murine model. However, the therapeutic potential and underlying mechanisms of DMM against obstructive nephropathy have not been investigated.

**Methods:**

We utilized the unilateral ureteral obstruction (UUO) mouse model to investigate the therapeutic potential of DMM in obstructive nephropathy. Histology, renal fibrosis, and inflammation were analyzed. A murine tubular cell line was used to investigate molecular mechanisms.

**Results:**

DMM administration mitigated UUO-induced renal fibrosis. Transcriptome analysis revealed that DMM promoted mitochondrial function and inhibited renal inflammation in UUO kidneys. The upregulated genes in DMM-treated mice were enriched in metabolic pathways related to fatty acids, organic acids, amino acids, and the PPAR signaling. DMM suppressed the accumulation of CD4^+^ T cells and the production of inflammatory cytokines in UUO kidneys. Moreover, DMM reduced oxidative stress by decreasing mitochondrial ROS production in tubular cells. Mechanistically, at least in part, DMM activated the PPAR signaling pathway in tubular cells, thereby enhancing fatty acid oxidation (FAO) activity and mitochondrial function. Pharmacological activation of PPAR protected against UUO-induced kidney fibrosis and inflammation.

**Conclusion:**

Our study suggests that targeting SDH with DMM could be a promising therapeutic strategy for obstructive nephropathy.

## 1 Introduction

Obstructive nephropathy is a leading cause of renal injury and fibrosis, which can be caused by kidney stones, prostatic hyperplasia, tumors, and congenital urinary abnormalities such as ureteropelvic junction obstruction (Chevalier, 2015; Stevens, 2018; Vemulakonda, 2021). Ureteral obstruction elevates intrapelvic and intratubular hydrostatic pressure and subsequently induces tubular cell damage and chronic inflammation in the kidney. The pro-fibrotic factors lead to the activation of myofibroblasts, which contribute to the excessive deposition of extracellular matrix and renal fibrosis (Humphreys, 2018; Jackson et al., 2018). Currently, the therapeutic options for obstruction-mediated renal fibrosis are limited.

Tubular cells are abundant in mitochondria because of the high energy demands required for maintaining normal kidney function, including electrolyte, water, and acid-base balance (Emma et al., 2016). Mounting evidence has demonstrated that mitochondrial dysfunction and metabolic aberrations are the hallmarks of both acute (Tran et al., 2016; Aparicio-Trejo et al., 2020; Wang Y. et al., 2021) and chronic kidney diseases in patients (Qi et al., 2017; Padovano et al., 2018; Fontecha-Barriuso et al., 2022). Notably, studies, including our own, have shown that obstructive nephropathy is characterized by mitochondrial dysfunction (Martinez-Klimova et al., 2020; Tao et al., 2023). In a murine model, unilateral ureteral obstruction (UUO) induces an early and sustained reduction in oxidative phosphorylation activity and mitochondrial mass (Jimenez-Uribe et al., 2021). Moreover, defective mitochondria contribute to the overproduction of reactive oxygen species (ROS) in the obstructed kidneys, triggering oxidative stress, kidney damage, and fibrosis (Aranda-Rivera et al., 2021). Consequently, the removal of damaged mitochondria by mitophagy restricts mitochondrial ROS (mtROS) generation and alleviates renal fibrosis induced by UUO (Li et al., 2020). An alternative strategy is to restore and boost mitochondrial function, but the options for suitable targets and compounds remain limited.

Renal tubule cells primarily rely on mitochondrial fatty acid oxidation (FAO) as their main energy source (Gao and Chen, 2022). Growing evidence suggests a link between renal pathogenesis and impaired FAO processes (Noels et al., 2021). In kidney diseases, key regulators of FAO (such as PPARα, CPT1A, and ACOX1) are frequently downregulated, exacerbating renal injury and fibrosis (Noels et al., 2021). For instance, PPARα inhibition disrupts FAO homeostasis and promotes fibrotic progression in UUO models (Li et al., 2022). Conversely, overexpression of renal tubule CPT1A, a rate-limiting enzyme in FAO, alleviates UUO-induced renal fibrosis by restoring mitochondrial homeostasis (Miguel et al., 2021). Thus, targeting the FAO process represents a promising therapeutic strategy for obstructive nephropathy.

Succinate dehydrogenase (SDH) plays a central role in mitochondrial metabolism (Dalla Pozza et al., 2020). It is an enzyme that catalyzes the oxidation of succinate to fumarate in the tricarboxylic acid (TCA) cycle and also serves as complex II of the electron transport chain in mitochondria (Van den Bossche et al., 2017). However, under hypoxic conditions, SDH operates reversely to generate succinate, a known inflammatory signal and contributor to ROS production (Chouchani et al., 2014; Martin et al., 2019). Dimethyl malonate (DMM), a malonate ester prodrug, acts as a potent SDH inhibitor (Beach et al., 2020). Although DMM has been shown to ameliorate renal ischemia-reperfusion injury by preserving mitochondrial function (Beach et al., 2020; Carlstrom et al., 2024), its precise mechanisms remain unclear. Furthermore, the therapeutic potential of DMM in obstructive nephropathy remains unexplored.

In this study, we investigated the therapeutic effects of SDH inhibition via DMM to elucidate whether and how DMM protects against kidney injury in obstructive nephropathy.

## 2 Materials and methods

### 2.1 Mice and UUO model

The C57BL/6 background mice were from GemPharmatech (Nanjing, China) and were maintained under specific pathogen-free conditions. The animal experiments were approved by the Ethical Committee of Quanzhou First Hospital. Male mice aged 8 weeks were used to establish the UUO model. A midline abdominal incision was performed after anesthesia (Avertin, Sigma, T48402) and the left ureter was double ligated. Mice were sacrificed 14 days post-UUO and kidneys were harvested for analysis. DMM (Sigma, 136,441, 40 mg/kg) was injected intraperitoneally every other day after UUO. Succinate (Sigma, 4%) was supplemented in the drinking water for 14 days after UUO.

### 2.2 Western blots

Kidney protein extracts were prepared according to standard protocols. Briefly, tissue lysates were separated by SDS-PAGE and transferred to polyvinylidene difluoride membranes (Millipore). The following primary antibodies were used: anti-goat KIM1 (R&D, AF1817-SP), anti-αSMA (Proteintech, 67,735-1-lg), anti-Col I (Abcam, ab260043), anti-fibronectin polyclonal antibody (Proteintech, 15613-1-AP), anti-cleaved caspase-3 (CST, 9661S), anti-IKK-β (CST, 2678T), anti-NF-κB p65 (CST, 8242T), anti-CD38 (Santa Cruz, sc-374650), anti-PPARα (Huaxingbio, HX18360), anti-PPARγ polyclonal antibody (Proteintech, 16643-1-AP), anti-β-actin (Huaxingbio, HX 1827), and anti-GAPDH (60004-1-Ig, Proteintech). Quantification was performed using ImageJ.

### 2.3 Masson staining and histological analysis

The mouse kidneys were fixed in 4% paraformaldehyde and embedded in paraffin. The kidney sections were stained with Masson’s trichrome. Images of the kidney slides were acquired using a NanoZoomer Slide Scanner (Hamamatsu Photonics). Quantification was performed using ImageJ software.

### 2.4 Succinate detection

The mouse kidneys were harvested and homogenized, followed by centrifugation at 10,000 rpm for 5 min. The supernatants were then analyzed for succinate levels using a Succinate Colorimetric Assay Kit (MAK184, Sigma) according to the manufacturer’s instructions.

### 2.5 Kidney leukocyte isolation and flow cytometry analysis

Mice were anesthetized with Avertin (Sigma, T48402). Kidneys were dissected into small fragments and digested in HBSS supplemented with 0.05% collagenase IV and 2 mM CaCl_2_ at 37°C for 25 min. Tissue homogenates were then filtered through a 70-μm nylon mesh and centrifuged at 500 *g* for 5 min. The resultant cell suspensions were treated with an Fcγ receptor-blocking antibody (101,320, BioLegend) for 10 min. The following fluorescently labeled antibodies (all from BioLegend) were applied: CD45-BV421 (103,134), CD11b-FITC (101,206), Ly6G-APC/Cyanine7 (127,624), Ly6C-PE (128,008), F4/80-APC (123,116), CD3-PE (100,206), CD4-PE/Cyanine7 (116,016), CD8a-APC/Cyanine7 (100,713), NK1.1-FITC (156,508), and CD20-APC (152,107). Flow cytometry was performed on a FACSCanto II (BD Biosciences). The data were analyzed using FlowJo software 10.4.

### 2.6 Real-Time quantitative PCR (qPCR)

The mouse kidneys were homogenized and total RNA was extracted using an RNA Extraction kit according to the manufacturer’s instructions (Huaxingbio, HXR8075). Complementary DNA was generated with a Reverse Transcription kit (Takara, RR037A). Real-time quantitative PCR was performed using the iCycler iQ5 Real-Time PCR detection system (Bio-Rad). The expression of the target genes was normalized to the expression of *Gapdh*. Relative gene expression was calculated using the standard 2^−ΔΔCT^ method. The following primers were used.

**Table:** 

Genes	Forward	Reverse
*Gapdh*	AGG​TCG​GTG​TGA​ACG​GAT​TTG	TGT​AGA​CCA​TGT​AGT​TGA​GGT​CA
*Col1a2*	GCA​GGT​TCA​CCT​ACT​CTG​TCC​T	CTT​GCC​CCA​TTC​ATT​TGT​CT
*Acta2*	ACT​GCC​GAG​CGT​GAG​ATT​GT	TGA​TGC​TGT​TAT​AGG​TGG​TTT​CG
*Fn*	CCC​TAT​CTC​TGA​TAC​CGT​TGT​CC	TGC​CGC​AAC​TAC​TGT​GAT​TCG​G
*Il1b*	TGT​AAT​GAA​AGA​CGG​CAC​ACC	TCT​TCT​TTG​GGT​ATT​GCT​TGG
*Il6*	CTG​CAA​GTG​CAT​CAT​CGT​TGT​TC	CTG​CAA​GTG​CAT​CAT​CGT​TGT​TC
*Tnf*	TCCAGGCGGTGCCTATGT	CAC​CCC​GAA​GTT​CAG​TAG​ACA​GA
*Ppara*	ACC​ACT​ACG​GAG​TTC​ACG​CAT​G	GAA​TCT​TGC​AGC​TCC​GAT​CAC​AC
*Pparg*	GTA​CTG​TCG​GTT​TCA​GAA​GTG​CC	ATC​TCC​GCC​AAC​AGC​TTC​TCC​T
*Cpt1a*	GGC​ATA​AAC​GCA​GAG​CAT​TCC​TG	CAG​TGT​CCA​TCC​TCT​GAG​TAG​C
*Acox1*	GCC​ATT​CGA​TAC​AGT​GCT​GTG​AG	CCG​AGA​AAG​TGG​AAG​GCA​TAG​G

### 2.7 Cell culture

The murine proximal tubular cell line TCMK-1 was obtained from Cell Resource Center of the Institute of Basic Medical Sciences (Beijing, China). The cells were cultured in DMEM supplemented with 10% fetal bovine serum. To induce epithelial-myofibroblast transition (EMT), the tubular cells were starved for 24 h and then treated with recombinant human TGF-β (MedChemExpress, HY-P7118, 10 ng/mL) either alone or in combination with DMM (2 mM) for 3 days.

### 2.8 Mitochondrial ROS (mtROS) detection

TCMK-1 cells were treated with TGF-β, DMM, or a combination of both agents as mentioned above. To detect mtROS, a MitoSOX Red Detection Kit (Beyotime, China, S0061S) was used according to the manufacturer’s instructions.

### 2.9 RNA sequencing

Bulk RNA sequencing was performed using UUO kidney tissues treated with PBS or DMM (n = 5). Total RNA was extracted from kidney tissues using an RNA Extraction kit according to the manufacturer’s instructions (Huaxingbio, HXR8075). RNA was qualified and quantified using a NanoDrop and an Agilent 2,100 bioanalyzer. Then RNA was amplified and reverse-transcribed to cDNA for library construction. Samples were sequenced on a NovaseqX Plus platform (Novogene, China). The sequencing data were aligned to the mouse reference genome (version mm10). Differentially expressed genes were analyzed using DESeq2 with five independent replicates using default parameters. Gene Ontology (GO) and Kyoto Encyclopedia of Genes and Genomes (KEGG) analyses were performed to determine the effect of DMM on the pathogenesis of obstructive nephropathy.

### 2.10 Statistical analysis

Statistical analyses were performed using GraphPad Prism version 6.0c. The data are presented as mean ± SEM. All experiments were replicated at least three times. The number of biological replicates is indicated in the figure legends. The two-tailed Student’s t-test was used for comparisons between two groups. One-way ANOVA was used to analyze three or more comparisons. A p-value less than 0.05 was considered significant.

## 3 Results

### 3.1 DMM inhibited UUO-induced renal fibrosis and injury

**FIGURE 1:**
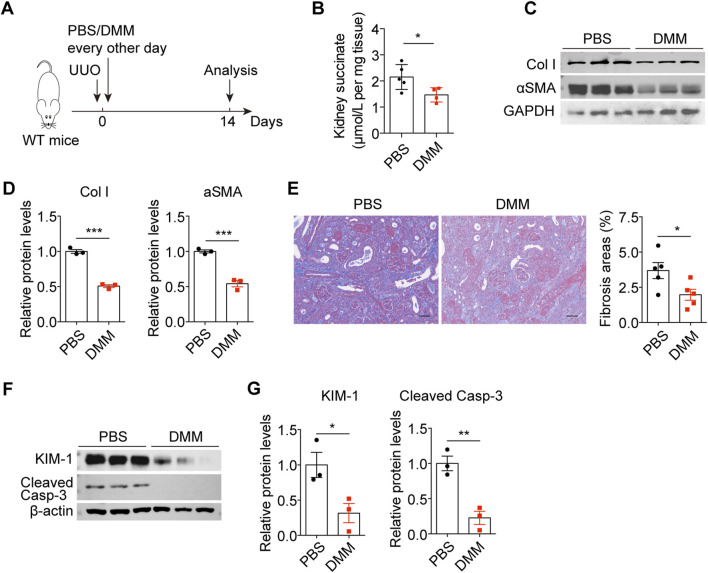
DMM inhibited UUO-induced renal fibrosis and injury. **(A)** The schematic of the experimental design. **(B)** The succinate content in PBS- and DMM-treated UUO kidneys (n = 5). **(C)** Immunoblots and **(D)** quantification of Col I and αSMA in UUO kidneys from mice treated with or without DMM (n = 3). **(E)** Representative Masson staining and quantification of UUO kidneys from mice treated with or without DMM (n = 5). **(F)** Immunoblots and **(G)** quantification of KIM1 and cleaved caspase-3 in UUO kidneys from mice treated with or without DMM (n = 3). The results represent mean ± SEM. *P < 0.05, **P < 0.01, ***P < 0.001.

To investigate the therapeutic effect of DMM on obstructive nephropathy, the UUO mouse model was used. UUO mice were treated with PBS or DMM every other day (Figure 1A). As previously reported, DMM administration reduced renal succinate content (Figure 1B). Importantly, DMM-treated mice exhibited decreased levels of collagen I (Col I) and α-smooth muscle actin (αSMA) in UUO kidneys compared to controls (Figures 1C,D). Histological analysis revealed that DMM administration resulted in a reduction in collagen-positive areas compared to the PBS-treated mice (Figure 1E). Moreover, DMM suppressed obstruction-induced renal injury and apoptosis, as evidenced by reduced expression of KIM1 and cleaved caspase-3 in UUO kidneys (Figures 1F,G). These results demonstrate that DMM attenuates UUO-induced renal fibrosis and injury.

### 3.2 DMM induced metabolic reprogramming and attenuated inflammation in UUO kidneys

**FIGURE 2:**
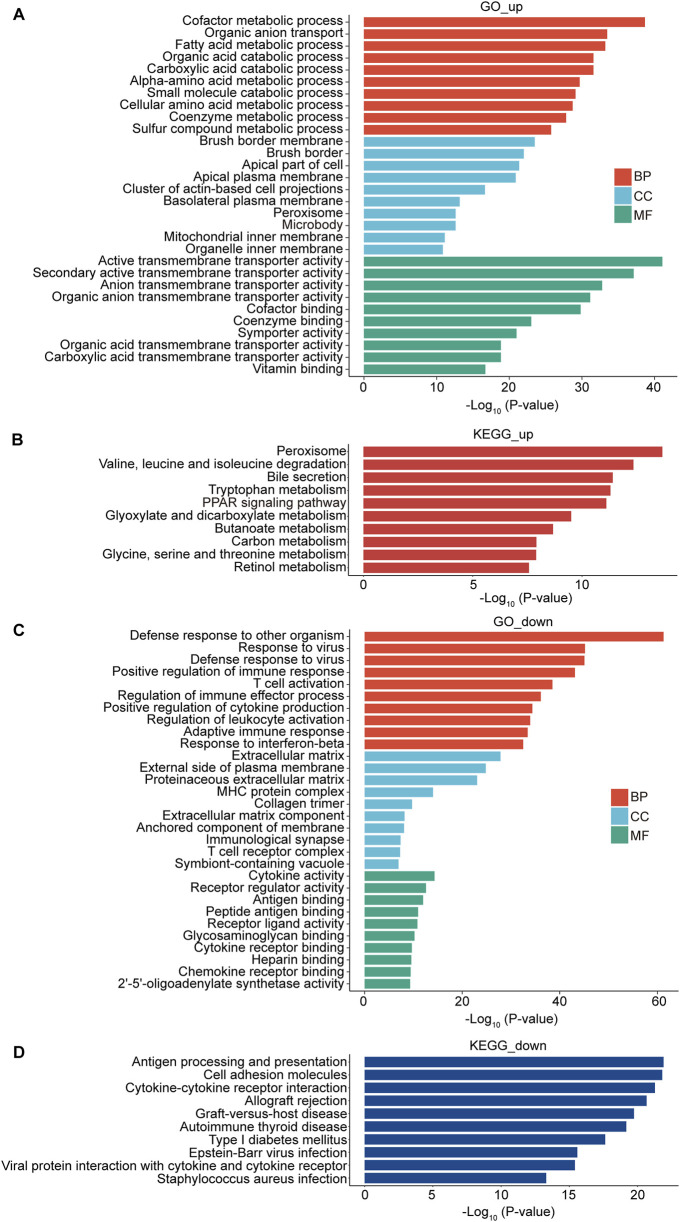
DMM triggered metabolic reprogramming and blunted inflammation in UUO kidneys. Bulk RNA-seq analysis of the UUO kidneys from mice treated with or without DMM. **(A)** GO analysis of the upregulated genes in DMM-treated mice. The top 10 enriched pathways were shown, including biological process (BP), cellular component (CC), and molecular function (MF). **(B)** KEGG analysis of the upregulated genes in DMM-treated mice and the top 10 pathways were shown. **(C)** GO analysis of the downregulated genes in DMM-treated mice and the top 10 enriched pathways were shown. **(D)** KEGG analysis of the downregulated genes in DMM-treated mice and the top 10 pathways were shown.

To investigate the mechanisms by which DMM regulates obstructive nephropathy, we performed bulk RNA sequencing (RNA-seq) of UUO kidneys treated with PBS or DMM. Gene Ontology (GO) analysis revealed that the upregulated genes (fold change >1.5) in DMM-treated kidneys were significantly enriched in metabolic pathways (Figure 2A). Specifically, biological processes (BP) involved in the metabolism of fatty acids, organic acids, amino acids, and coenzymes were enriched in DMM-treated kidneys (Figure 2A). Cellular component (CC) analysis revealed enrichment of upregulated genes in brush border and mitochondrial inner membranes (Figure 2A). In addition, Kyoto Encyclopedia of Genes and Genomes (KEGG) analysis identified significant enrichment of metabolic pathways, including peroxisome metabolism, tryptophan metabolism, and PPAR signaling activation (Figure 2B). These transcriptomic findings collectively indicate that DMM treatment enhances metabolic homeostasis in UUO kidneys. Importantly, our findings align with previous studies indicating that mitochondrial dysfunction and metabolic aberrations are central pathological features of chronic kidney diseases, including obstructive nephropathy (Fontecha-Barriuso et al., 2022; Tao et al., 2023).

Conversely, the downregulated genes (fold change >1.5) in DMM-treated kidneys were significantly enriched in pro-inflammatory biological processes, such as positive regulation of immune response, T cell activation, positive regulation of cytokine production, and adaptive immune response (Figure 2C). Molecular function (MF) enrichment showed that the downregulated genes were involved in cytokine activity, antigen binding, and receptor-ligand activity (Figure 2C). KEGG analysis demonstrated a notable association of the downregulated genes with immune response, such as antigen processing and presentation, cell adhesion molecules, and cytokine-cytokine receptor interaction (Figure 2D). Taken together, these findings suggest that DMM exerts dual therapeutic effects by simultaneously enhancing metabolic function and suppressing inflammatory cascades in obstructed kidneys.

### 3.3 DMM inhibited the production of mtROS in tubular cells

**FIGURE 3:**
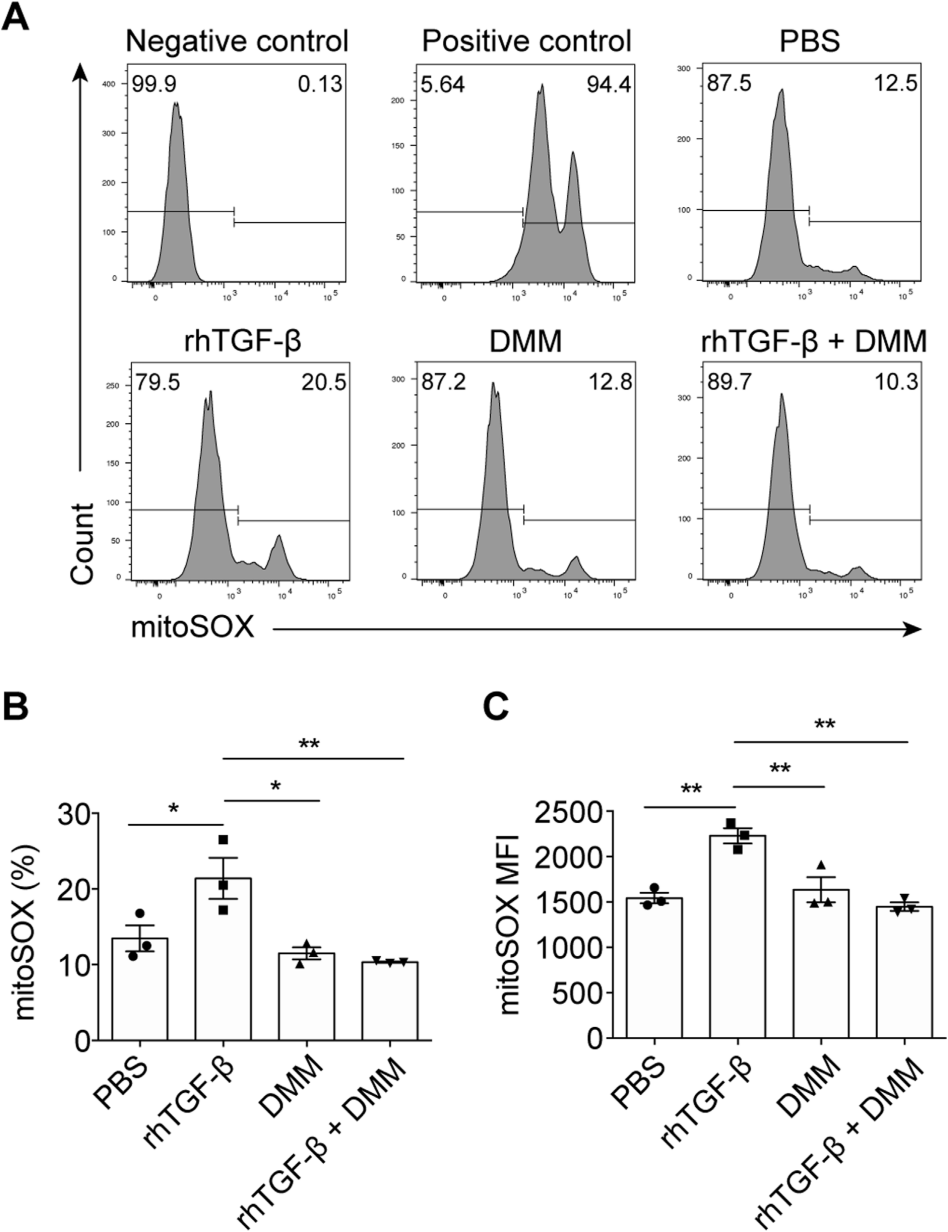
DMM inhibited the production of mtROS in tubular cells. **(A)** Representative flow cytometry plots of MitoSOX Red in TCMK-1 cells treated with positive stimulation, rhTGF-β, DMM, and rhTGF-β + DMM. **(B)** Quantification of MitoSOX Red positive cells in **(A)** (n = 3). **(C)** Quantification of mean fluorescence intensity (MFI) of MitoSOX Red in TCMK-1 cells (n = 3). The results represent mean ± SEM. *P < 0.05, **P < 0.01.

Oxidative stress, a key feature of obstructive nephropathy, is consistently associated with mitochondrial dysfunction (Aranda-Rivera et al., 2021). Given that DMM enhanced mitochondrial function in UUO kidneys, we investigated whether mtROS was affected by DMM. Through *in vitro* experiments using the murine tubular cell line TCMK-1, we found that TGF-β stimulation induced mtROS generation during tubular cell epithelial-myofibroblast transition (EMT) (Figures 3A–C), consistent with previous reports (Veith et al., 2021; Liao et al., 2022). Notably, DMM administration almost completely abolished mtROS production in TCMK-1 cells (Figures 3A–C). These data demonstrate that DMM effectively inhibits mtROS production in tubular cells.

### 3.4 DMM inhibited CD4^+^ T cell infiltration and kidney inflammation

**FIGURE 4:**
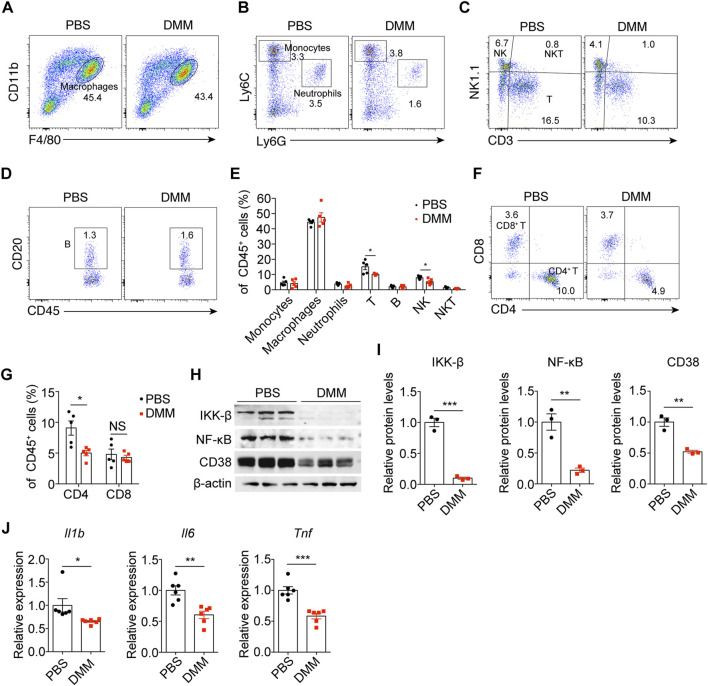
DMM inhibited CD4^+^ T cell infiltration and kidney inflammation. Representative flow cytometry plots of **(A)** macrophages, **(B)** monocytes and neutrophils, **(C)** NK, NKT, and T cells, and **(D)** B cells in UUO kidneys from mice treated with or without DMM. **(E)** Quantification of the indicated immune cells in PBS- and DMM-treated UUO kidneys (n = 5). **(F)** Representative flow cytometry plots and **(G)** quantification of CD4^+^ and CD8^+^ T cells in UUO kidneys from mice treated with or without DMM (n = 5). **(H)** Immunoblots and **(I)** quantification of IKK-β, NK-κB p65, and CD38 in UUO kidneys from mice treated with or without DMM (n = 3). **(J)** qPCR analysis for IL-1β, IL-6, and TNF expression in PBS- or DMM-treated UUO kidneys (n = 6). The results represent mean ± SEM. *P < 0.05, **P < 0.01, ***P < 0.001.

To investigate the effects of DMM on renal inflammation, we analyzed kidney immune cell populations by flow cytometry. Interestingly, DMM administration did not alter the accumulation of monocytes, macrophages, neutrophils, B cells, or natural killer T (NKT) cells in obstructed kidneys (Figures 4A–E). However, compared to the control mice, DMM-treated mice exhibited significantly reduced infiltration of CD4^+^ T cells and natural killer (NK) cells into UUO kidneys (Figures 4C,E–G), consistent with RNA-seq findings. Immunoblot analysis further demonstrated that DMM significantly suppressed NF-κB pathway activation (Figures 4H,I). We have previously shown that CD38 is an important promoter of renal inflammation in UUO kidneys (Tao et al., 2023). Interestingly, DMM administration significantly decreased CD38 expression in obstructed kidneys (Figures 4H,I). Additionally, the inflammatory cytokines IL-1β, IL-6, and TNF-α exhibited a significant downregulation in DMM-treated mice compared to those in PBS-treated mice (Figure 4J). Collectively, these results indicate that DMM inhibits inflammation in UUO kidneys.

### 3.5 DMM promoted PPAR signaling in UUO kidneys and tubular cells

**FIGURE 5:**
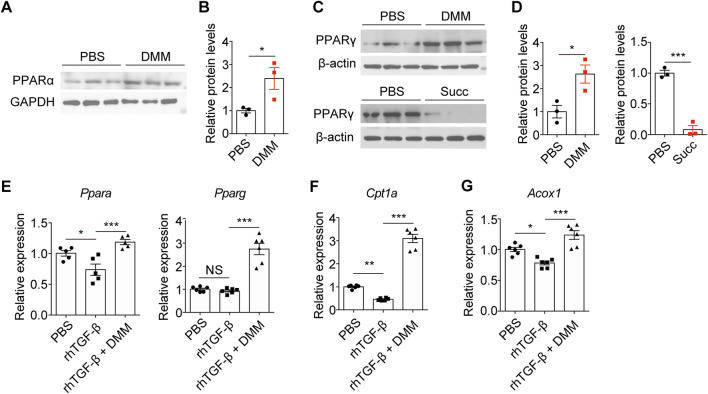
DMM promoted PPAR signaling in UUO kidneys and tubular cells. Immunoblots and quantification of PPARα **(A,B)** and PPARγ **(C,D)** in UUO kidneys from mice treated with DMM or succinate (n = 3). **(E)** qPCR analysis for PPARα and PPARγ expression in TCMK-1 cells treated rhTGF-β or rhTGF-β + DMM (n = 5–6). qPCR analysis for **(F)** *Cpt1a* and **(G)** *Acox1* in TCMK-1 cells treated rhTGF-β or rhTGF-β + DMM (n = 6). The results represent mean ± SEM. *P < 0.05, **P < 0.01, ***P < 0.001, NS no significance.

PPAR signaling is central to fatty acid metabolism and mitochondrial function (Lyu et al., 2018). RNA-seq analysis revealed that DMM treatment activated PPAR signaling. Immunoblots showed that DMM-treated mice exhibited significant upregulation of both PPARα and PPARγ in UUO kidneys (Figures 5A–D). To counteract the DMM-induced reduction in succinate levels, exogenous succinate was administered via drinking water. This intervention abolished the DMM-mediated upregulation of PPARγ (Figures 5C,D), confirming succinate’s regulatory role in this pathway. In TCMK-1 cells, TGF-β stimulation downregulated PPARα expression but showed no significant effect on PPARγ (Figure 5E), suggesting selective suppression of PPAR signaling during TGF-β-mediated EMT. Notably, DMM promoted the expression of both PPARα and PPARγ, especially PPARγ (Figure 5E). Furthermore, CPT1α and ACOX1, key rate-limiting enzymes in FAO, exhibited same expression patterns as PPAR in the presence of DMM (Figures 5F,G). Taken together, these data demonstrate that DMM promotes PPAR signaling in tubular cells.

### 3.6 Pharmacological activation of PPAR signaling protected against UUO-induced kidney fibrosis and inflammation

**FIGURE 6:**
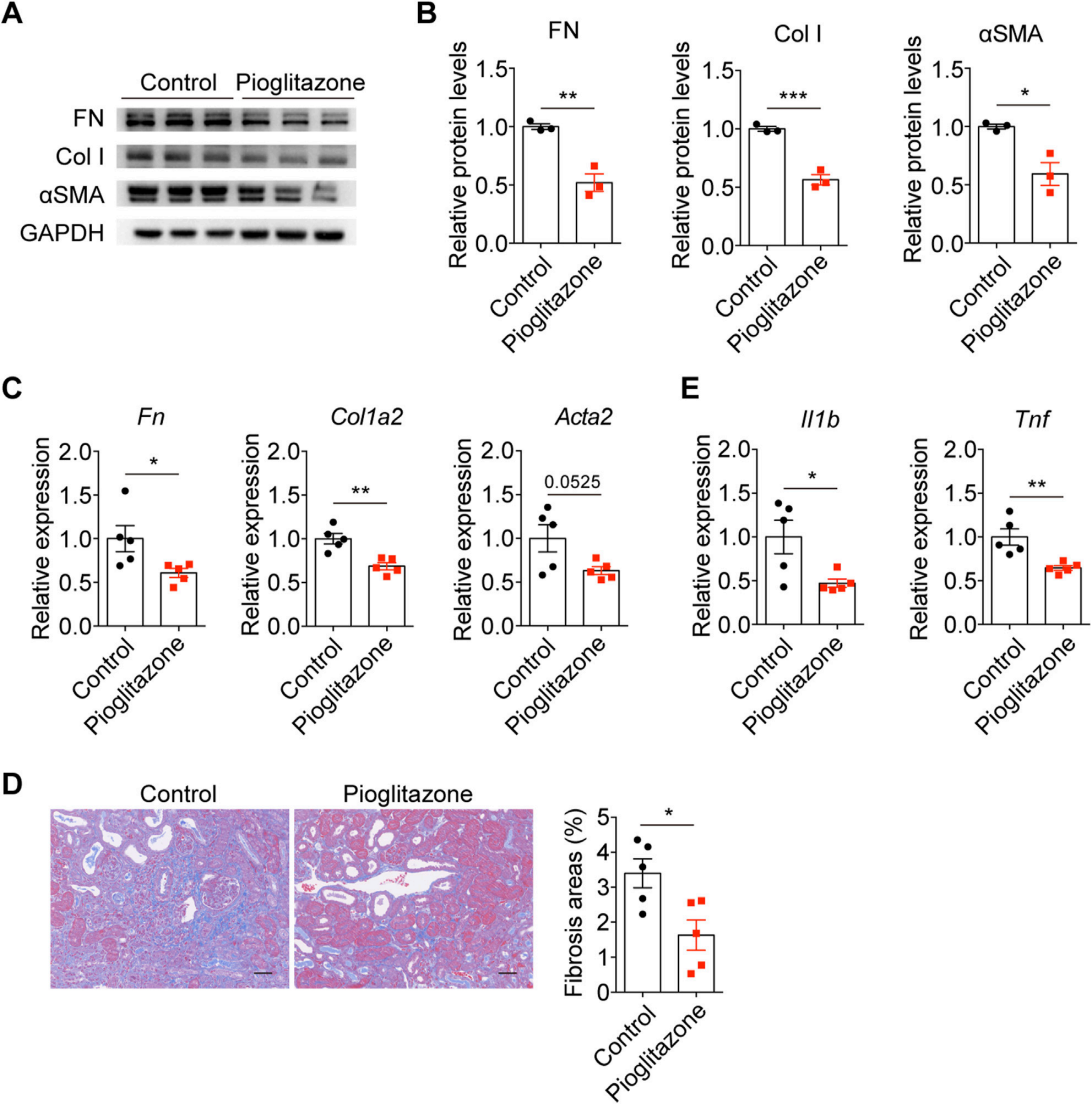
Pharmacological activation of PPAR protected against UUO-induced kidney fibrosis and inflammation. **(A)** Immunoblots and **(B)** quantification of FN, Col I, and αSMA in UUO kidneys from mice treated with or without pioglitazone (n = 3). **(C)** qPCR analysis for FN, Col I, and αSMA in UUO kidneys from mice treated with or without pioglitazone (n = 5). **(D)** Representative Masson staining and quantification of UUO kidneys from mice treated with or without pioglitazone (n = 5). **(E)** qPCR analysis for IL-1β and TNF in UUO kidneys from mice treated with or without pioglitazone (n = 5). The results represent mean ± SEM. *P < 0.05, **P < 0.01, ***P < 0.001.

To further investigate the therapeutic potential of PPAR signaling activation, we administered the PPAR agonist pioglitazone in the UUO model. Pioglitazone treatment significantly suppressed the expression of fibronectin (FN), Col I, and αSMA at both protein and mRNA levels in UUO kidneys (Figures 6A–C). Histological analysis demonstrated that PPAR activation substantially reduced collagen deposition, as evidenced by decreased collagen-positive areas in obstructed kidneys (Figure 6D). Furthermore, pioglitazone-treated mice exhibited significantly lower levels of pro-inflammatory cytokines IL-1β and TNF-α in UUO kidneys compared to controls (Figure 6E). These findings collectively indicate that pharmacological activation of PPAR signaling exerts protective effects against both fibrotic and inflammatory processes in obstructive nephropathy.

## 4 Discussion

In this study, we showed that DMM effectively prevented obstruction-induced kidney injury and fibrosis. DMM administration promoted metabolic reprogramming in the kidney, partially through PPAR-mediated enhancement of fatty acid metabolism. Furthermore, DMM inhibited CD4^+^ T cell infiltration and mtROS production, thereby alleviating inflammation and oxidative stress in obstructed kidneys.

In obstructive nephropathy, mechanical stretching triggers activation of the renin-angiotensin system (RAS) through angiotensin II, thus activating nicotinamide adenine dinucleotide phosphate (NADPH) oxidases that produce ROS (Aranda-Rivera et al., 2021). Malfunctioning mitochondria are also significant sources of ROS in renal disorders (Zhang et al., 2021). Oxidative stress causes alterations in pathways related to apoptosis and inflammation, contributing to the development of renal injury and fibrosis (Aranda-Rivera et al., 2021). ROS overproduction induces tubular cell apoptosis by activating caspase-3, ERK1/2, and p38 (Rhyu et al., 2005; Ostergaard et al., 2014). Moreover, excessive ROS are linked to the activation of TGF-β and NF-κB, key players in myofibroblast activation and inflammation, respectively (Iglesias-De La Cruz et al., 2001; Nakatani et al., 2002). Our study demonstrated that DMM alleviated mitochondrial dysfunction and reduced mtROS production in tubular cells. Additionally, DMM administration decreased the levels of NF-κB p65 and the injury marker KIM1 in UUO kidneys. In fact, previous studies have suggested that enhancing mitochondrial function can alleviate obstruction-induced kidney injury and fibrosis (Aranda-Rivera et al., 2022; Liao et al., 2022).

PPARs are nuclear receptors that function as ligand-activated transcription factors, including three isoforms: PPARα, PPARβ/δ, and PPARγ (Wagner and Wagner, 2020). The involvement of PPARα-mediated FAO has been implicated in various renal conditions, such as acute kidney injury, diabetic nephropathy, and chronic kidney disease (Gao and Gu, 2022). Notably, there is a growing body of evidence showing that the activation of PPARγ confers a protective effect against renal interstitial fibrosis (Gao and Gu, 2022). Clinical studies have demonstrated the therapeutic potential of PPARγ agonists in reducing albuminuria in patients with type 2 diabetes (Pistrosch et al., 2012). Beyond metabolic regulation, PPARγ exhibits anti-inflammatory properties by modulating macrophage polarization. Specifically, PPARγ activation promotes the differentiation of anti-inflammatory M2 macrophages, thereby attenuating tissue inflammation (Chen et al., 2019; Kokeny et al., 2021). In our study, DMM treatment significantly enhanced PPAR signaling pathways, although the precise mechanisms underlying this interaction require further investigation.

One of the primary pathogenic mechanisms in obstructive nephropathy involves impaired renal perfusion caused by elevated intratubular hydrostatic pressure (Jackson et al., 2018). This renal hypoperfusion creates a hypoxic microenvironment where SDH operates in reverse to produce succinate from fumarate (Spinelli et al., 2021). We revealed that DMM-mediated SDH inhibition led to an increase in renal succinate levels in UUO kidneys. Studies have shown that succinate is associated with lung, liver, and renal fibrosis by driving myofibroblast activation and M2 macrophage polarization (Li et al., 2015; Liu et al., 2020; Wang Z. et al., 2021; Pu et al., 2024). Notably, our results revealed that exogenous succinate supplementation suppressed PPAR signaling, suggesting its potential role in exacerbating metabolic dysregulation in obstructed kidneys. Therefore, DMM-mediated SDH inhibition may mitigate fibrosis by reducing succinate accumulation and restoring PPAR signaling.

Interestingly, DMM administration inhibited renal inflammation and CD4^+^ T cell accumulation. This effect may be attributed to DMM-mediated metabolic reprogramming that alters the tissue microenvironment, thereby modulating the inflammatory response. Notably, succinate serves as a key inflammatory signal capable of inducing CD4^+^ T cell activation through mechanisms associated with enhanced chromatin accessibility of pro-inflammatory transcription factors (Chen et al., 2022). Therefore, the anti-inflammatory properties of DMM may be related to its ability to reduce succinate levels, although the specific mechanisms require further investigation.

Clinically, beyond surgical interventions, targeted pharmacotherapy for obstructive nephropathy remains lacking. Our findings establish DMM as a therapeutic agent capable of targeting mtROS production, metabolic reprogramming, and inflammation in preclinical models of obstructive nephropathy. However, human chronic obstruction exhibits slower progression compared to murine acute models, which necessitates dose optimization in larger mammals and biomarker-driven patient stratification. Of note, DMM administration demonstrated sustained anti-fibrotic effects at 14 days post-UUO. Nevertheless, the investigation of both the long-term efficacy and safety profile of DMM will be essential to strengthen its therapeutic potential.

## 5 Conclusion

In conclusion, DMM administration inhibits kidney inflammation, mtROS production, and UUO-induced renal injury and fibrosis, partially via promoting PPAR signaling pathway. Our findings suggest that DMM-mediated SDH inhibition could serve as a therapeutic strategy for obstructive nephropathy.

## Data Availability

The datasets presented in this study can be found in online repositories. The names of the repository/repositories and accession number(s) can be found below: https://www.ncbi.nlm.nih.gov/geo/, GSE278764.
